# Long-term saline water irrigation affected soil carbon and nitrogen cycling functional profiles in the cotton field

**DOI:** 10.3389/fmicb.2024.1310387

**Published:** 2024-03-14

**Authors:** Shuang Zhou, Guangshuai Wang, Qisheng Han, Junpeng Zhang, Hongkai Dang, Huifeng Ning, Yang Gao, Jingsheng Sun

**Affiliations:** ^1^Institute of Farmland Irrigation of Chinese Academy of Agriculture Sciences, Ministry of Agriculture and Rural Affairs, Xinxiang, China; ^2^Graduate School of Chinese Academy of Agricultural Sciences, Beijing, China; ^3^College of Water Conservancy and Civil Engineering, Shandong Agricultural University, Taian, China; ^4^Key Laboratory of Crop Drought Resistance Research of Hebei Province/Institute of Dryland Farming, Hebei Academy of Agriculture and Forestry Sciences, Hengshui, China; ^5^Western Agricultural Research Center, Chinese Academy of Agricultural Sciences, Changji, China

**Keywords:** soil metagenomics, saline water irrigation, carbon cycle, nitrogen cycle, functional genes

## Abstract

Saline water irrigation (SWI) plays an important role in alleviating water resource shortages. At the same time, the salt input of irrigation water affects soil microorganisms which participate in various ecological processes of terrestrial ecosystems. However, the responses of soil microbial functional potential to long-term SWI remains unclear. Therefore, Metagenomics method was utilized in cotton fields under long-term SWI to reveal the microbial functional profiles associated with soil carbon and nitrogen cycles. Results indicated that SWI impacted the microbial functional profiles of soil carbon and nitrogen cycles in the cotton fields significantly. Especially, irrigation water salinity inhibited the relative abundances of *sacC* and *vanB*, which are soil carbon degradation genes. SWI also affected the functional gene abundance of nitrogen degradation, dissimilatory nitrate reduction, and nitrification. Moreover, SWI significantly increased the abundance of *Candidatus_Cloacimonetes* in both carbon and nitrogen cycles. In the discussion, we used person analysis found that soil salinity, pH, and ammonium nitrogen were important factors affecting the abundance of functional genes and microbial taxa. Overall, this study indicated that long-term SWI significantly influenced specific microbial functional genes and taxa abundance, which may lead to predictable outcomes for soil carbon and nitrogen cycling, and is of great importance in exploring the impact of SWI on soil environments.

## 1 Introduction

Soil carbon (C) and nitrogen (N) cycles is the conversional process of soil C and N between different forms (Luo et al., 2020), which are important for soil quality, affecting the productivity and stability of the agroecological system (Shah et al., 2020). It is well known that soil microbes are the drivers of soil C and N cycles (Du et al., 2023). Therefore, studying microbial functional profiles changes related to soil C and N cycling is crucial for improving soil quality and agricultural system productivity.

Reduced rainfall and the scarcity of freshwater lead to soil moisture deficits and decreased crop yields (Han et al., 2023). It is necessary to find a way to alleviate those questions to ensure the stability of agricultural production. Utilizing saltwater resources judiciously has been demonstrated feasible (Lew et al., 2020). Nevertheless, saline water irrigation (SWI) brings salt into the soil. Some studies found salt could affect microbial processes of soil C and N cycles (Poffenbarger et al., 2011; Yu et al., 2019; Shahariar et al., 2021). Our previous research has also found that SWI alters the content of soil environmental factors involved in C and N cycles, such as soil organic matter, inorganic N, and greenhouse gasses (Zhou et al., 2023, 2024). The changes in soil environmental factor content are crucial for assessing soil quality and health. In order to safely utilize SWI, it is necessary for us to explore the impact of SWI on soil microbial processes involved in C and N cycling.

Soil microbial processes are orchestrated by microorganisms through the activation of functional genes (Zhang et al., 2021; Shi et al., 2023). The study results about how salt affects the C cycle genes are inconsistent. Mamilov et al. (2004) demonstrated that salinity can inhibit soil C cycle functional gene abundance, while Morrissey et al. (2014) showed that salinity significantly enhanced C cycle genes. A similar phenomenon emerges in N cycle analysis (Tang et al., 2012; Cui et al., 2016). It was found that elevated soil salinity markedly suppresses the prevalence of denitrification and nitrification genes (Li et al., 2012; Wang et al., 2018), while some studies showed SWI could increase the abundance of denitrification genes (Guo et al., 2023). The soil microbial genes involved in C and N cycles are abundant, and their response to salt is intricate. So the effect of salinity on different functional genes remains further investigated. In addition, Microbial taxa have received extensive attention as performers of functional genes. Yang et al. (2023) indicated that soil salinity significantly reduced soil C cycling-related microbial diversity. Guo et al. (2023) suggested that SWI significantly reduced the abundances of *Proteobacteria* and *Acidobacteria*, while simultaneously boosting the abundances of *Gemmatimonadetes*, *Actinobacteria*, and *Chloroflexi.* Previous findings suggested that the varied responses of distinct soil C and N cycle microbial taxa to salt stress (Zhang et al., 2023). It is imperative to give greater consideration to soil microbial taxa under SWI.

Previous studies have indicated a strongly correlation between soil microbial functional genes and taxa abundance with soil environmental factors (Cui et al., 2016; Guo et al., 2023; Xiang et al., 2023). Loganathachetti et al. (2022) proposed that soil salinity was an important determinant of bacterial richness and shannon diversity index. Shen et al. (2016) discovered that the bacterial taxonomic diversity/structure was strongly influenced by soil pH. Sun et al. (2021) found that some special microbial taxa abundance was significantly correlated with nitrate N, ammonium N and total N content. Furthermore, Luo et al. (2020) and Suter et al. (2021) suggested that there was a close coupling relationship between the functional genes of C and N cycles. In order to explore the reasons for the change of microbial profiles under SWI, it is essential to explore the connection between microorganisms and soil properties, and the correlation of genes between the two cycles. However, few studies examined the relationships between microorganisms and soil properties, as well as the relationships between C and N cycles. It is imperative to undertake the aforementioned research in the long-term SWI field.

Based on our previous findings indicating that SWI alters the content of soil environmental factors involved in C and N cycles, we hypothesize that SWI changes the microbial functional profile of soil C and N cycling, leading to the aforementioned outcomes. In this study, we utilized metagenomics and statistical techniques to quantify the abundance of functional genes and microbial taxa linked to soil C and N cycling. The objectives of this study encompassed four main aspects: (i) investigating the impact of SWI on functional genes related to soil C and N cycles, (ii) pinpointing the key microbial taxa harboring characteristic genes related to soil C and N cycles, (iii) developing connections between functional genes, microbial taxa, and soil properties, and (iv) evaluating the interaction among these genes in soil C and N cycling.

## 2 Materials and methods

### 2.1 Experimental setup and soil sampling

The experiment was conducted at the experimental station of Hebei Province Academy of Agriculture and Forestry Sciences, China. The climate in this area is a warm temperate continental monsoon climate, and the soil is loam. Three treatments were set up in the 15-year SWI experiment: irrigation of local groundwater (SWI1: 1 g L^−1^), irrigation of 4 g L^−1^ salt water (SWI4), and irrigation of 8 g L^−1^ salt water (SWI8). Border irrigation was used for the study. In addition, cotton exhibits robust salt tolerance and drought resistance, making it a frequent choice as an initial crop during SWI (Wang et al., 2022). More details about the experimental setup can be found in Zhou et al. (2023) and Supplementary Table S1.

Soil samples were collected at the 0–20 cm depth in July 2021. Soil was taken according to the S method and mixed into one sample at each plot. From each treatment, three composite samples were gathered. The soil samples were employed for the extraction of DNA and the measurement of soil properties. In this study, we evaluated soil salinity (EC*_e_*), pH, ammonium N, nitrate N, and organic matter. Soil salinity was measured using a conductivity meter (Orion Star A322, Thermo Scientific, United States) (Zhang et al., 2020). Soil pH was measured using a pH probe (Orion Star A321, Thermo Scientific, United States) (Shen et al., 2016). Soil nitrate N and ammonium N were measured by a flow analyzer (Thermo, VarioskanFlash, United States) (Gao et al., 2013). Soil organic matter was determined by the low-temperature external thermal potassium dichromate oxidation-colorimetric method (Cao et al., 2011). Details regarding the techniques used for measuring these soil characteristics can be found in Supplementary Text S1.

### 2.2 DNA extraction and sequencing

**Figure 1:**
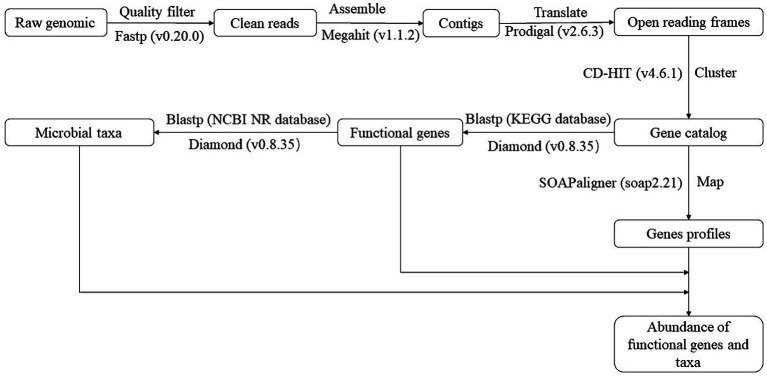
The flow of metagenomic sequence data processing. KEGG was the Kyoto Encyclopedia of Genes and Genomes. NCBI was the National Center for Biotechnology Information. NR database was the Non-Redundant protein sequence database.

DNA was extracted and measured by FastDNA^®^ SPIN Kit (MP Biomedicals, United States) and NanoDrop 2000 spectrophotometer, respectively. The information on soil DNA was transmitted to the Majorbio Bio-pharm Technology Co., Ltd. (Shanghai, China) for metagenomics sequencing. The sequencing was conducted in an Illumina NovaSeq 6000 platform (Illumina Inc., San Diego, CA, United States). The Sequence raw data can be found in the National Center for Biotechnology Information (NCBI) with accession NO PRJNA1018300.^1^ Figure 1 illustrates the flow of metagenomic sequence data processing. Supplementary Table S2 shows the basic sequence information. Supplementary Table S3 provides the KEGG orthology number, name, and encoded protein of functional genes. The relative abundance of functional genes was calculated using Reads Per Kilobase Million (Lawson et al., 2017) for statistical analysis.

### 2.3 Statistical analysis

The statistical analyses were performed utilizing R language (v4.0.4). To elucidate variations in microbial functional gene groups across various treatments, PCoA (principal coordinate analysis) and ANOSIM (analysis of similarities) was applied, utilizing the Bray-Curtis distance. We employed the Kruskal-Wallis test to ascertain notable dissimilarities in functional genes and microbial taxa (with relative abundances exceeding 0.1%) among the various treatments. We utilized Spearman correlation analysis to evaluate the association between soil properties and genes/taxa. The associations were visualized in heatmaps. The co-occurring network was constructed by Gephi^2^ to show the relationship between functional genes of the two cycles.

## 3 Results

### 3.1 SWI affects the functional groups of soil C and N cycling

**Figure 2:**
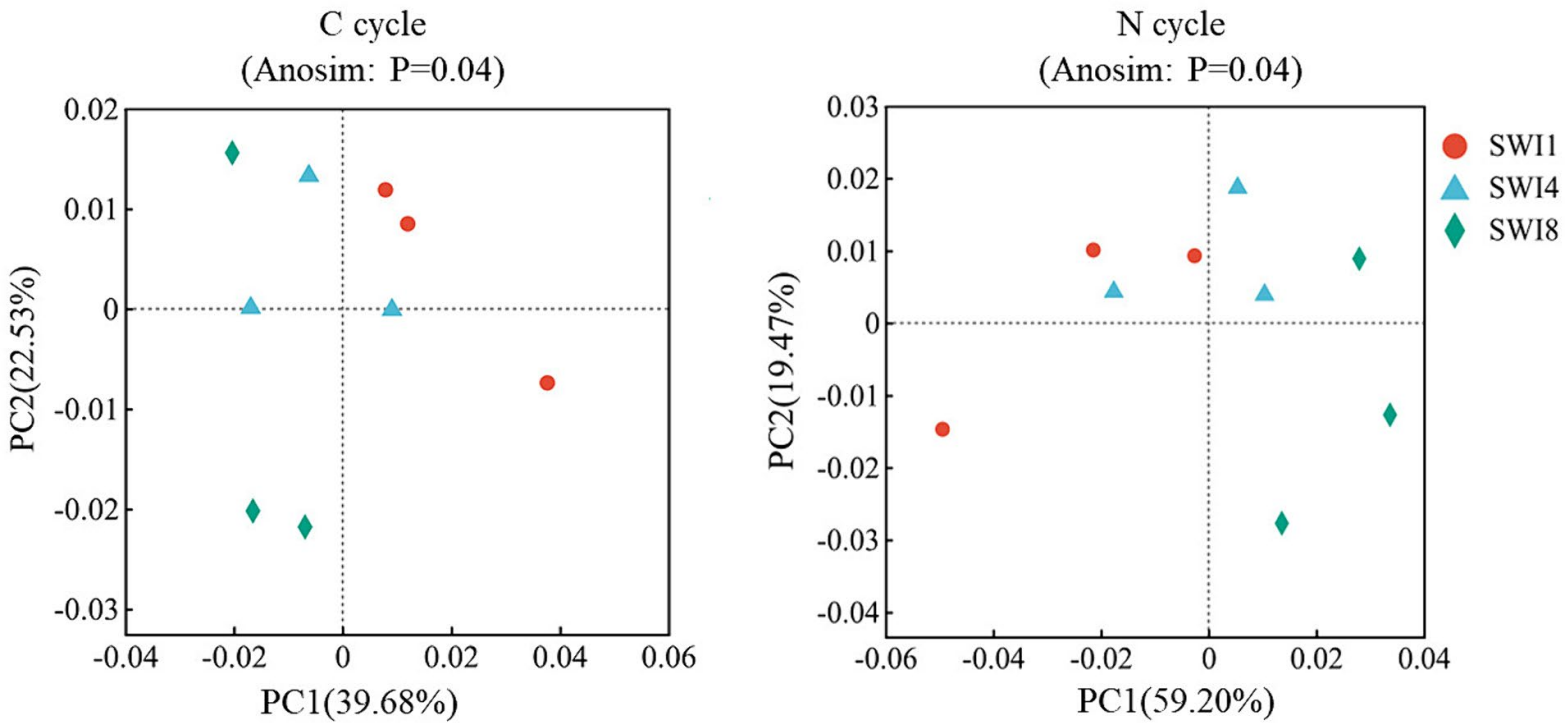
PCoA analysis and ANOSIM analysis of carbon and nitrogen cycling functional genes in saltwater-irrigated cotton fields. C is carbon. N is nitrogen. PCoA analysis was used to determine microbial functional profiles in the soil carbon and nitrogen cycle. ANOSIM analysis was used to determine differences in microbial functional profiles between treatments.

Fifty eight functional genes related to the soil C cycle and 37 genes related to the N cycle were detected from all samples (Supplementary Table S3). The C cycle functional genes contains: C degradation (40 genes), C fixation (13 genes), and methane metabolism (5 genes). The N cycle functional genes contains: N fixation (2 genes), nitrification (4 genes), denitrification (8 genes), assimilatory nitrate reduction to ammonium (ANRA: 5 genes), dissimilatory nitrate reduction to ammonium (DNRA: 7 genes), N degradation (11 genes). The *p*-value of the significance difference after performing ANOSIM test was 0.04 for both the C and N cycles (Figure 2). SWI accounted for the majority of variations observed in functional profiles among treatments (PC1:39.68% for C cycle; PC1:59.20% for N cycle) (Figure 2). The results indicated that SWI changed the C and N cycling function of soil microorganisms. SWI significantly changes functional gene abundance of the soil C cycle.

### 3.2 SWI significantly changes functional gene abundance of the soil C cycle

**Figure 3:**
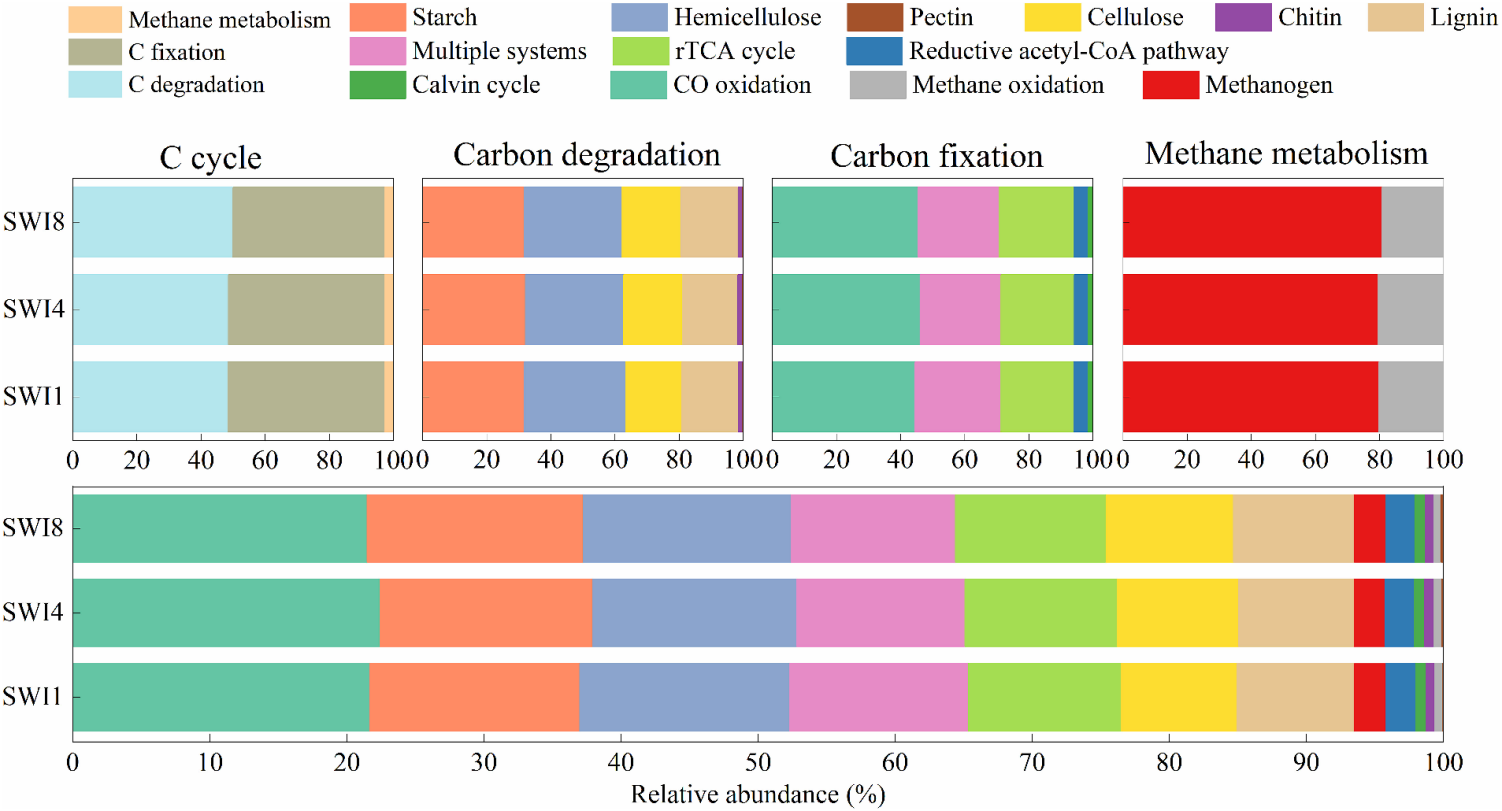
Relative abundance ratio of functional genes in each pathway of the soil carbon cycle. C is carbon. N is nitrogen. rTCA cycle is the reductive tricarboxylic acid cycle.

Functional genes of soil C cycle in this study are mainly divided into 3 categories: C degradation, C fixation, and methane metabolism (Supplementary Table S3). Starch and hemicellulose-degrading genes demonstrated a greater abundance than others among C-degrading genes (Figure 3). The rank of functional gene abundance among C fixation was CO oxidation > multiple systems > reductive tricarboxylic acid cycle (rTCA cycle) > reductive acetyl-CoA pathway > Calvin cycle. In methane metabolism, the abundance of methanogen genes exceeded that of methane oxidation (Figure 3). Compared to the SWI1, the C degradation gene abundance of SWI4 and SWI8 increased by 0.35 and 3.12%, the C fixation gene abundance of SWI4 and SWI8 decreased by 0.32 and 3.03%, and the methane metabolism gene abundance of SWI4 and SWI8 decreased by 0.52 and 1.11%.

**Figure 4:**
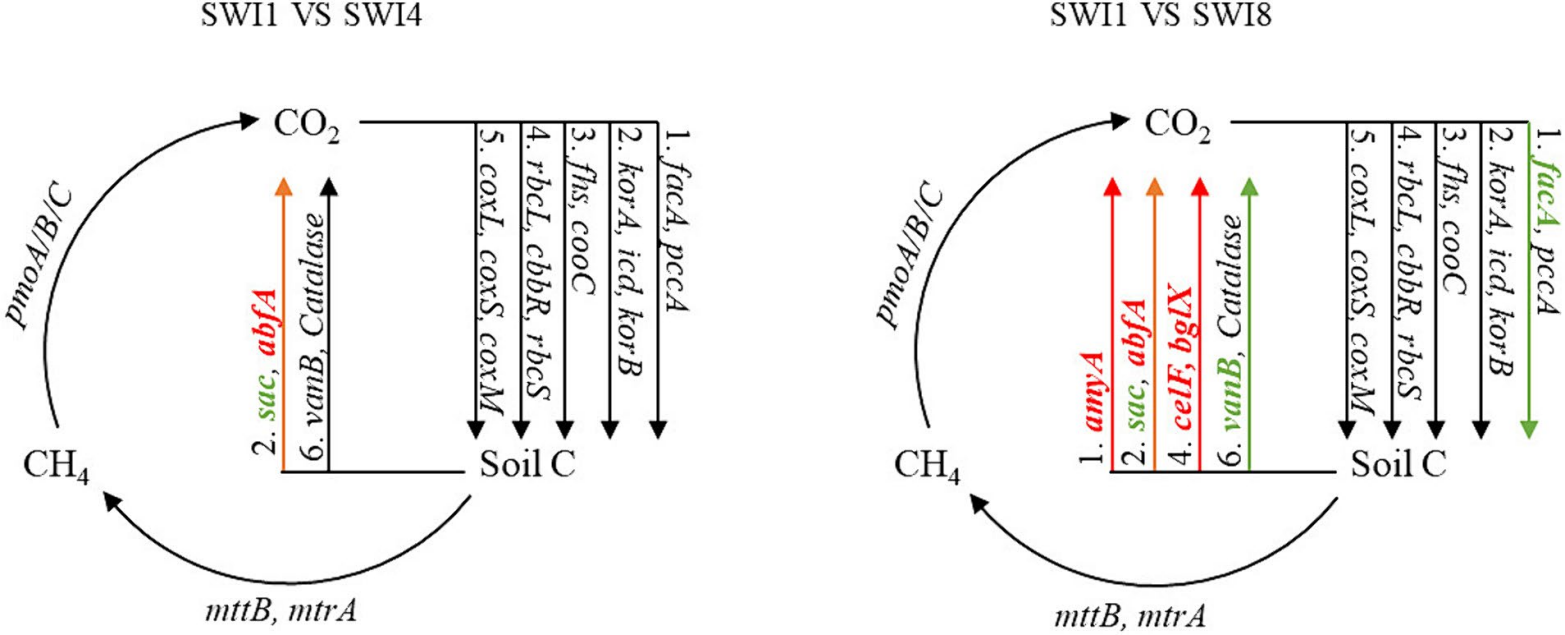
Diagram depicting the different carbon cycling processes based on metagenomic sequencing. SWI1: irrigation water salinity was 1 g L^−1^. SWI4: irrigation water salinity was 4 g L^−1^. SWI8: irrigation water salinity was 8 g L^−1^. Red indicates that the gene abundances were significantly and consistently increased in the latter treatment. Green indicates that the gene abundances were significantly and consistently decreased in the litter treatment. Black indicates no significant difference between the treatments.

**Figure 5:**
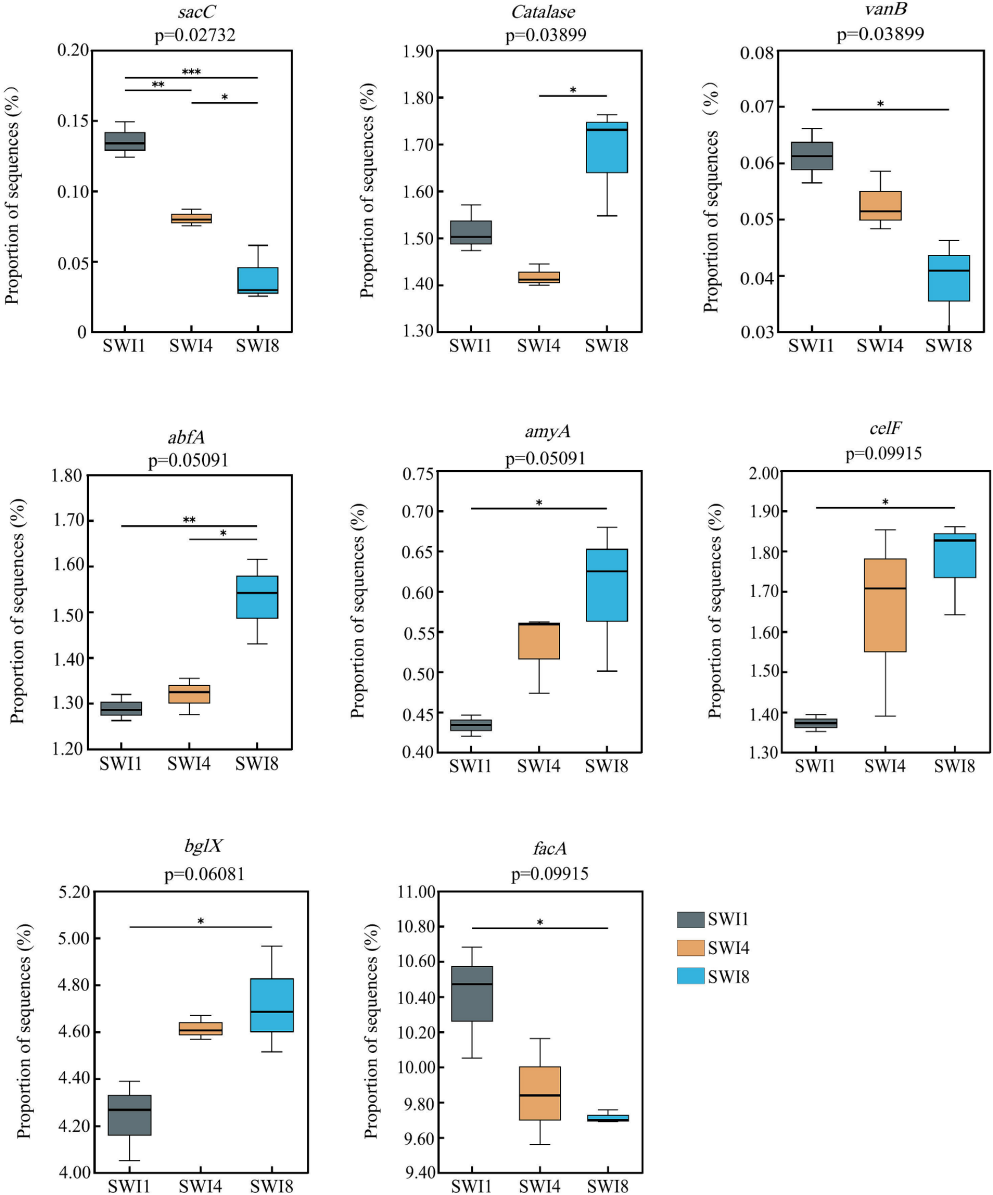
Carbon cycling function genes were significantly affected by saline water irrigation. SWI1: irrigation water salinity was 1 g L^−1^. SWI4: irrigation water salinity was 4 g L^−1^. SWI8: irrigation water salinity was 8 g L^−1^.

Kruskal-Wallis test indicated that SWI significantly affected C-degrading gene abundance (*sacC, Catalase*, and *vanB*) (Supplementary Figure S1). In particular, salinity significantly decreased the abundance of *sacC* (hemicellulose degradation) and *vanB* (lignin degradation). We also found that irrigation water with varying salinity levels exerted a notable influence on the abundance of diverse functional genes (Figure 4). Compared with SWI1, SWI4 decreased the abundance of *sacC* by 40.52% and increased *abfA* by 2.25%, respectively (Figure 5), the gene abundances of *sacC*, *vanB*, *facA* (C fixation) in SWI8 were significantly decreased by 71.45, 36.27, and 6.60%, respectively, while the gene abundances of *abfA*, *amyA* (starch degradation), *celF* (cellulose degradation), and *bglX* (cellulose degradation) were markedly increased by 18.62, 38.88, 29.45, and 11.45%, respectively (Figure 5). Furthermore, the abundance of *sacC, Catalase*, *and abfA* differed significantly between SWI4 and SWI8 (Figure 5).

### 3.3 Response of soil N cycle functional gene abundance to SWI

**Figure 6:**
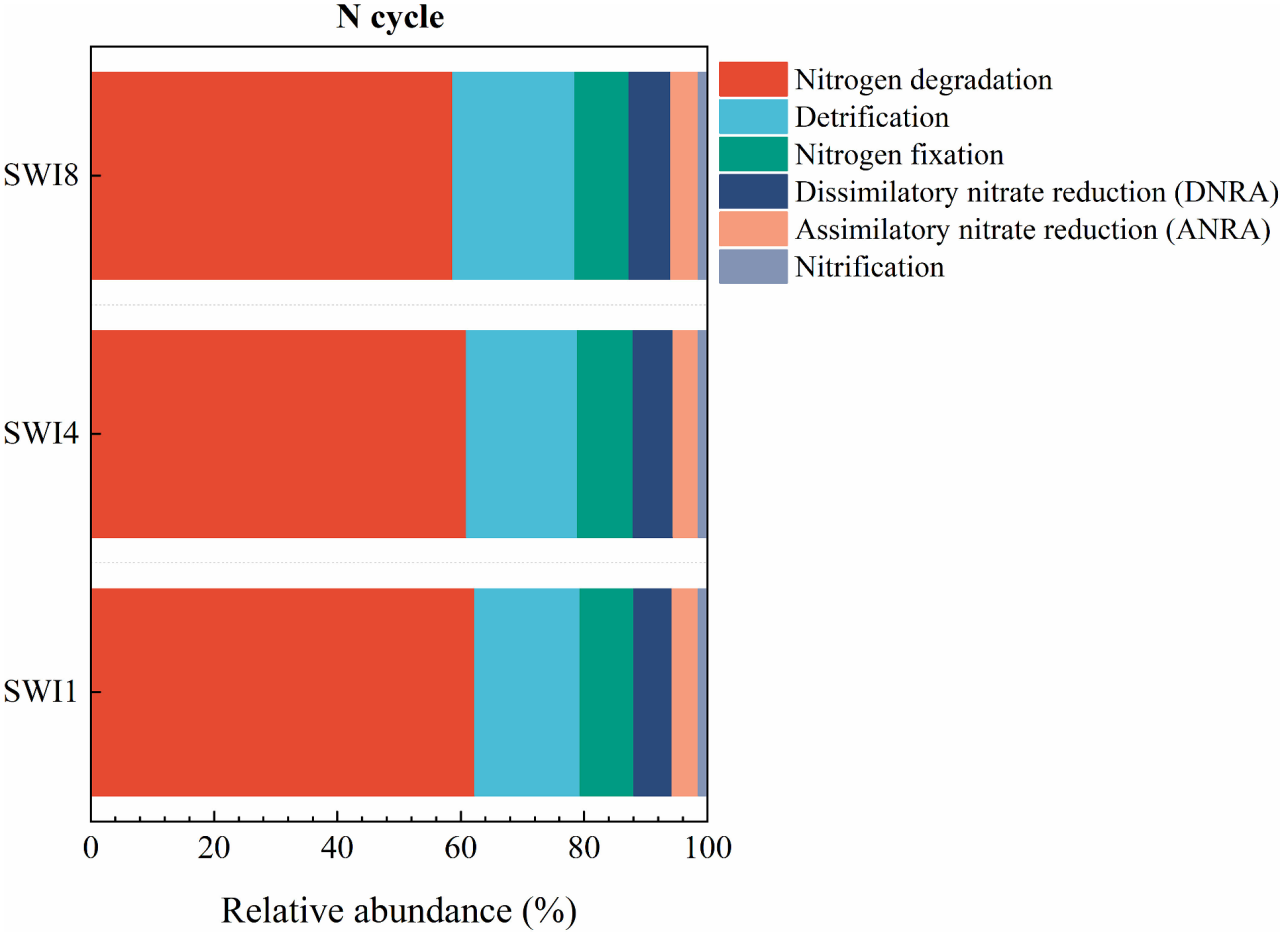
Relative abundance ratio of functional genes in each pathway of the soil nitrogen cycle. N is nitrogen. SWI1: irrigation water salinity was 1 g L^−1^. SWI4: irrigation water salinity was 4 g L^−1^. SWI8: irrigation water salinity was 8 g L^−1^.

**Figure 7:**
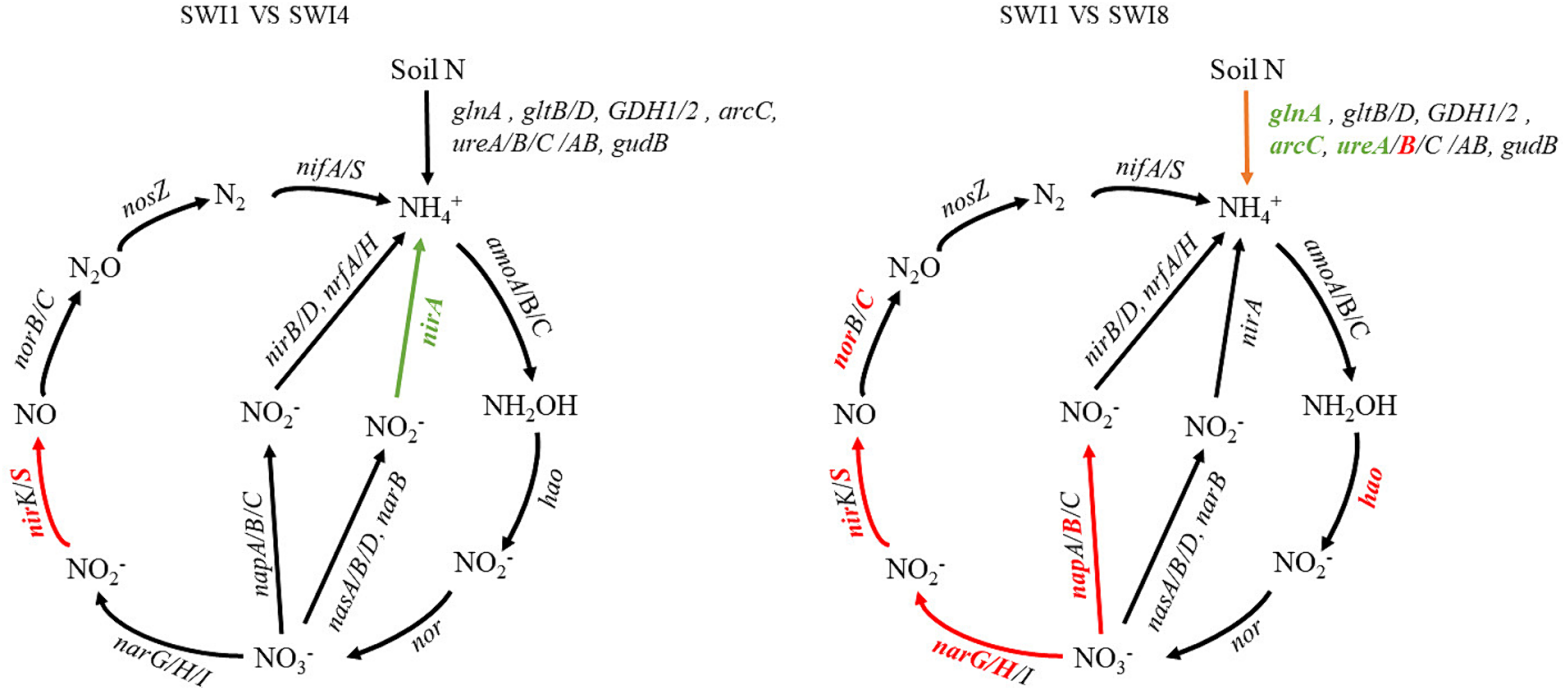
Diagram depicting the different nitrogen cycling processes based on metagenomic sequencing. N is nitrogen. SWI1: irrigation water salinity was 1 g L^−1^. SWI4: irrigation water salinity was 4 g L^−1^. SWI8: irrigation water salinity was 8 g L^−1^. Red indicates that the gene abundances were significantly and consistently increased in the latter treatment. Green indicates that the gene abundances were significantly and consistently decreased in the litter treatment. Black indicates no significant difference between the treatments.

**Figure 8:**
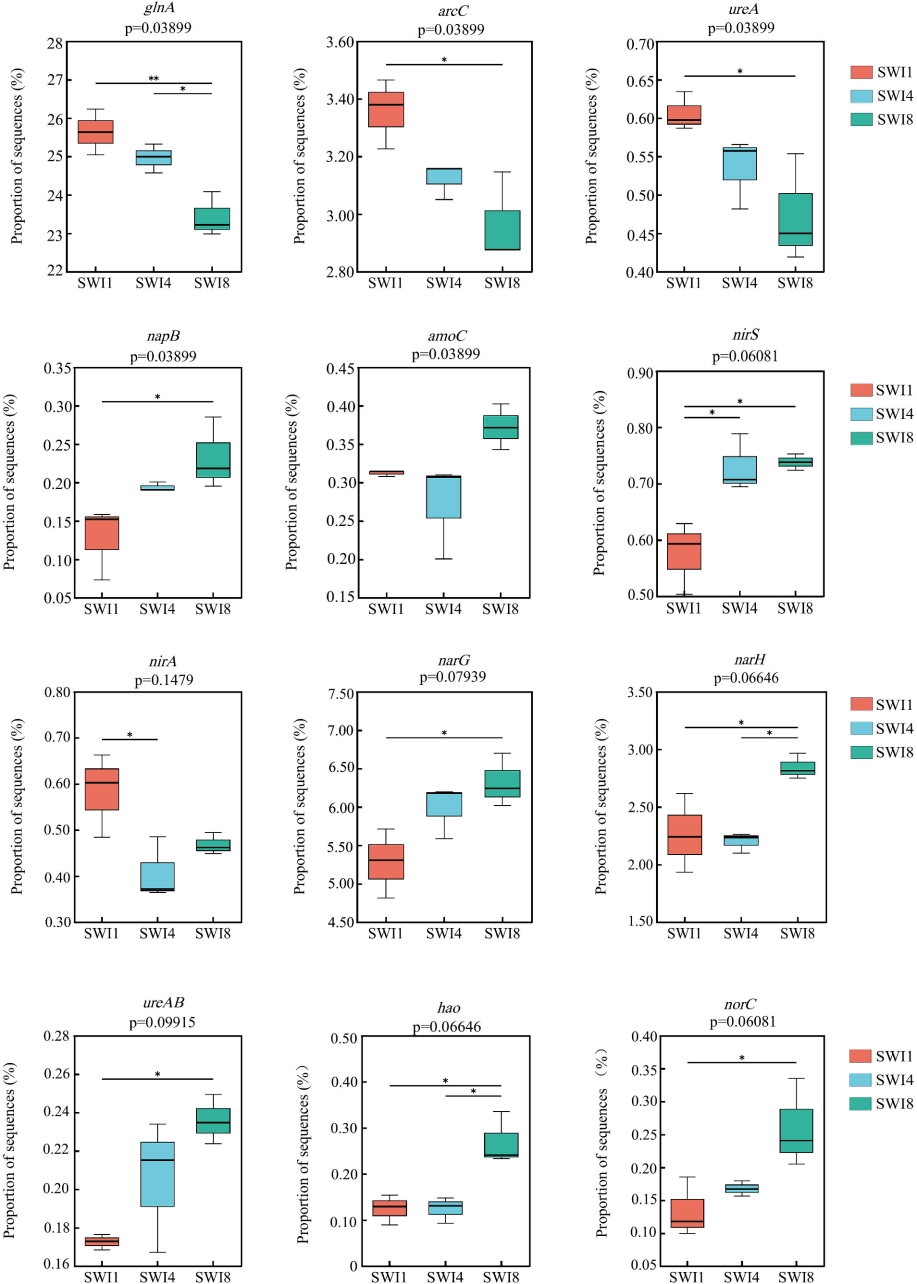
Nitrogen cycling function genes were significantly affected by saline water irrigation. SWI1: irrigation water salinity was 1 g L^−1^. SWI4: irrigation water salinity was 4 g L^−1^. SWI8: irrigation water salinity was 8 g L^−1^.

The soil N cycle genes include N fixation, denitrification, nitrification, ANRA, and DNRA in the study. N-degrading gene abundance was the highest, accounting for 58.72–62.32%. It was followed by denitrification, N fixation, DNRA, and ANRA (Figure 6). As the salinity of irrigation water increases, the abundance of soil N degradation genes gradually decreases, while the abundance of denitrification, N fixation, and DNRA genes gradually increases. Compared to SWI1, the N degradation gene abundance of SWI4 and SWI8 decreased by 2.19 and 5.78%, the denitrification gene abundance of SWI4 and SWI8 increased by 5.56 and 15.76%, the N fixation gene abundance of SWI4 and SWI8 increased by 3.89 and 2.05%, DNRA gene abundance of SWI4 and SWI8 increased by 4.12 and 8.30%. The abundance of ANRA and nitrification genes varied across different irrigation water salinity. Compared with SWI1, ANRA and nitrification gene abundance of SWI4 decreased by 3.63 and 1.31%, while the gene abundance of SWI8 increased by 3.99 and 3.05%. SWI significantly affected soil N degradation (*glnA, arcC, ureA*), DNRA (*napB*), and nitrification (*amoC*) gene abundance (Supplementary Figure S2). Among the genes, SWI significantly decreased the abundance of *glnA*, *arcC,* and *ureA*, while markedly increasing *napB* abundance. The impact of varying salt concentrations in irrigation water exhibited diverse effects on the functional attributes of N-cycling microorganisms (Figure 7). SWI4 significantly increased the gene abundance of *nirS* by 26.97% and decreased the gene abundance of *nirA* by 30.16% (Figure 8). SWI8 decreased the abundance of *glnA*, *arc*, and *ureA* by 8.58, 11.65, and 21.80%, respectively, (*p* < 0.05), while increased *napB*, *nirS*, *narG*, *narH*, *ureAB*, *hao*, and *norC* abundance by 82.30, 28.38, 19.75, 25.64, 36.67, 117.42, and 93.96%, respectively, (*p* < 0.05) (Figure 8). Moreover, the results also indicated *glnA, narH*, *and hao* abundance differed significantly between SWI4 and SWI8 (Figure 8).

### 3.4 Effects of SWI on soil C and N cycle microbial taxa

The predominant phyla associated with soil C cycling genes were *Actinobacteria*, *Proteobacteria*, *Acidobacteria*, and *Chloroflex* (Supplementary Figure S3). SWI affected the abundance of *Bacteroidete* and *Candidatus-Cloacimonetes* significantly (Supplementary Figure S4). Compared with SWI1, SWI4 and SWI8 increased the abundance of *Bacteroidete* (31.16 and 59.54%) and *Candidatus_Cloacimonetes* (120.30 and 434.37%) (Supplementary Figure S5).

*Actinobacteria* and *Proteobacteria* accounted for 59.16–60.72% within microbial taxa related to soil N cycle. In addition to the two dominant phyla, the abundance of *Acidobacteria*, *Thaumarchaeota*, and *Chloroflexi* was higher than other microbial taxa (Supplementary Figure S6). SWI significantly changed the abundance of *Candidatus-Cloacimonetes* (Supplementary Figures S7, S8). The abundance of *Candidatus_Cloacimonetes* increased significantly by 78.27% in SWI4 and by 236.38% in SWI8 compared with SWI1 (Supplementary Figure S8).

### 3.5 Correlations between functional profiles of soil microbes and environmental factors

Soil properties of the saltwater-irrigated cotton fields are provided in Supplementary Table S4. Correlation analysis results indicated a substantial impact of salinity on both C and N cycling in the soil (Supplementary Figure S9). There were negative correlations observed between soil salinity and C-degrading genes (*rfbB*, *MAN2C1*, *xylF*, *sacC*, and *bglA*), C fixation genes (*facA*, *pccA*, *rbcL*, and *coxM*), and N-degrading genes (*glnA*, *arcC*, *ureC*, and *ureA*), but positively correlated with denitrification (*narG*, *nirS*, and *norC*), DNRA (*napA* and *napB*), and nitrification (*hao*) genes. According to the heat map (Supplementary Figure S9), soil pH affected soil C degradation and fixation gene abundance significantly, with a negative correlation observed between *MAN2C1*, *facA,* and pH (*p* < 0.01). In addition, the denitrification genes (*narG* and *nirS*) were positively correlated with soil pH. Soil ammonium N was positively correlated with DNRA (*napB*) and negatively correlated with nitrification (*amoB*) (Supplementary Figure S9). Moreover, the abundance of *Proteobacteria* and *Bacteroidetes* was positively correlated with soil salinity, and the abundance of *Acidobacteria* was negatively correlated with soil salinity (Supplementary Figure S10).

### 3.6 Interaction among soil C and N cycling functional genes

**Figure 9:**
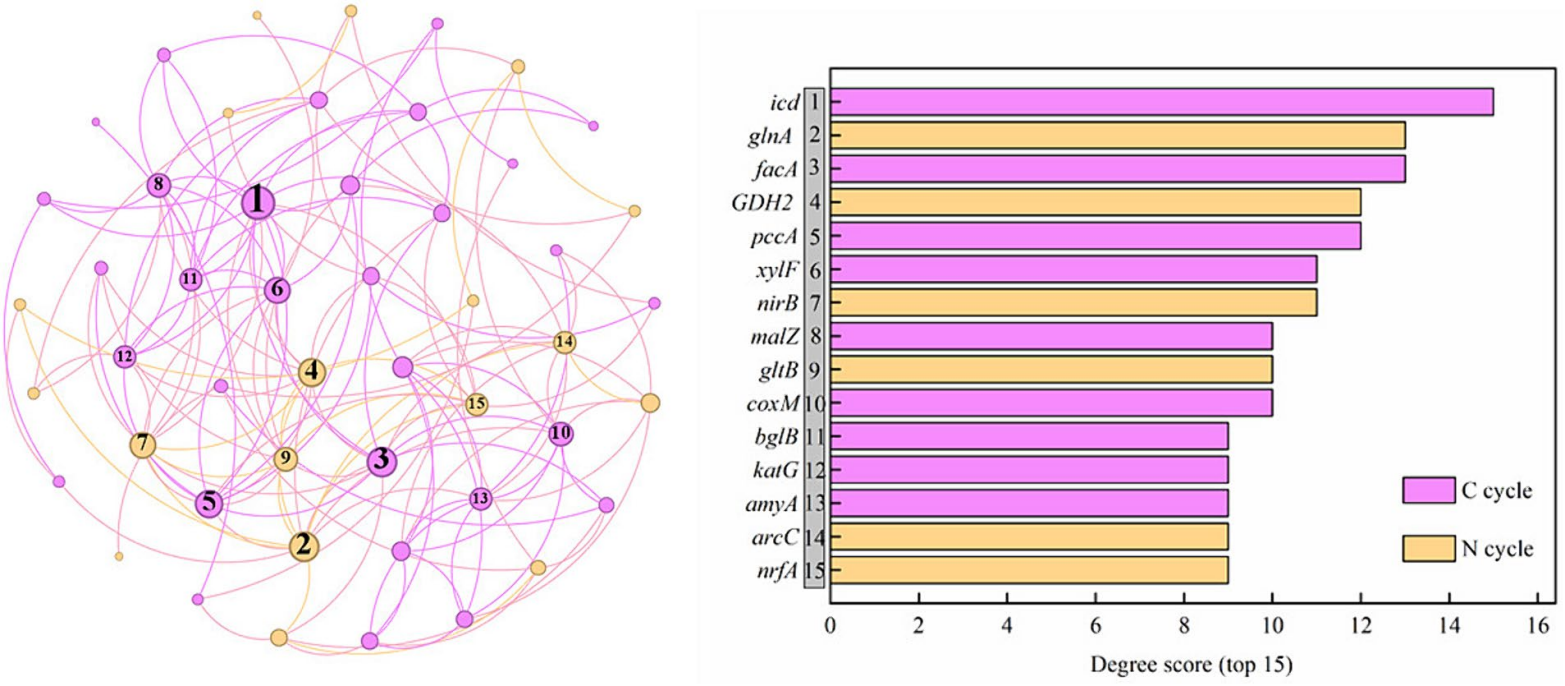
Network analysis between genes related to carbon and nitrogen cycling genes and degree scores for the networks (top 15). C is carbon. N is nitrogen.

Figure 9 shows a co-occurring network of the internal connections of functional genes during the C and N cycles. The top 15 genes ranked according to degree included 9 C-cycle Genes (*icd*, *facA*, *pccA*, *xylF*, *malZ*, *coxM*, *bglB*, *katG*, and *amyA*) and 6 N-cycle genes (*glnA*, *GDH2*, *nirB*, *gltB*, *arcC*, and *nrfA*). According to Spearman correlation analysis, there were complex relationships between C and N cycle genes. Among the top 15 genes, there was a notable positive correlation between functional genes of C fixation (*pccA*, *pccA*, *facA*, and *pccA*) and DNRA (*nirB*), soil N degradation (*GDH2*, *glnA*, and *gltB*). Additionally, a notable positive correlation existed between soil functional genes involved in C degradation (*xylF, katG, amyA*, and *katG*) and those related to denitrification (*narG* and *nirK*) and DNRA (*nirB* and *nirB*), while a notable negative correlation existed between *amyA*, *malL* (C degradation) and *arcC* (N degradation), *nrfA* (DNRA).

## 4 Discussion

### 4.1 SWI induced variations in soil functional gene groups in the cotton field

Previous studies have demonstrated that SWI has the potential to alter soil microbial communities and enzyme activities (Saviozzi et al., 2011; Haj-Amor et al., 2022). However, the impact of SWI on microbial functional gene groups remains unclear. This study revealed different SWI treatments have significant differences in the soil C and N functional gene groups through PCoA and ANOSIM analyses (Figure 2). This indicates that SWI significantly influences the composition of soil C and N functional genes. Du et al. (2023) indicated that changes in soil C and N cycling genes and microorganisms were closely related to soil properties. Hu et al. (2022) also found that variations in the soil environment may lead to disparities in microbial taxa and functional genes. In Section 3.5, we also observed this phenomenon in the correlation analysis between functional genes and soil environmental factors. The study found that changes in soil functional gene abundance were closely associated with soil salinity, pH value, and ammonium N content (Supplementary Figures S9, S10). To further explore the impact of SWI on the soil C and N cycling functional gene groups, we conducted comparative analysis of every soil C and N cycling functional gene abundance.

### 4.2 SWI altered the abundance of functional genes associated with C degradation and fixation

The functional genes of soil C degradation dominated the C cycle gene composition with a large proportion, which aligns with the outcomes observed in Du et al. (2023) and Hu et al. (2022). The reason why C degradation genes occupy a large proportion in soil is related to the characteristics of soil microorganisms and ecosystem environment (Ren et al., 2022). Kruskal-Wallis test in the study showed that SWI significantly reduced the abundance of C degradation genes *sacC* and *vanB*, which was consistent with earlier researches. The decrease in the abundance of C degradation functional genes due to salinity is associated with its reduction in soil microbial activity and biomass (Yan et al., 2015). However, not all C degradation genes significantly affected by SWI exhibit a trend of decreasing abundance with increasing irrigation salinity. In the multi-group comparison of soil C functional gene abundance, the soil C degradation gene *Catalase* was also significantly affected by SWI, with its abundance being lowest in the SWI4 treatment and highest in the SWI8 treatment, which may be because the selective decomposition of soil C sources by microbial communities in response to varying external conditions (Hu et al., 2022). Furthermore, both *VanB* and *Catalase* are functional genes related to lignin degradation, but their responses to SWI were different. This variation could be attributed to different C degradation functional genes responding differently to the soil environment (Jing et al., 2021). This situation also occurred in the results of a pairwise comparison between SWI1 and SWI4. In the comparison between SWI1 and SWI8, our investigation revealed that SWI8 markedly suppressed the presence of certain C degradation genes, while concurrently elevating the levels of genes associated with starch degradation (*amyA*) and cellulose degradation (*celF* and *bglX*). This may be because microorganisms need to promote the degradation of different C sources to maintain the element balance in the soil (Luo et al., 2019). In addition, SWI exerted a substantial impact on the process of soil C fixation. The abundance of *facA* in SWI8 was significantly lower than that in SWI1, which indicated that the ability of high-salinity soil to synthesize stable C compounds and store them in soil for a long time may be reduced.

### 4.3 SWI significantly affected the abundance of soil N cycle functional genes

SWI significantly affected soil N degradation (*glnA, arc,* and *ureA*), DNRA (*napB*), and nitrification (*amoC*) gene abundance. Highly active N degradation genes can promote NH_4_^+^ production which could be directly absorbed and utilized by crops (Geisseler et al., 2010). Soil microorganisms conversion of ammonia into glutamine (*glnA*) and the breakdown of urea into ammonia (*ureA*) can expedite the N cycle, thereby supplying nutrients for plant growth (Dixon et al., 1976; Bernard and Habash, 2009). However, the abundance of *glnA, arcC,* and *urea* declined as irrigation water salinity increased in the study, which indicated that SWI inhibited soil N degradation (Haj-Amor et al., 2022). This could be because soil salinity has the potential to restrict crucial N transformation processes by modifying microbial and soil physical and chemical characteristics (Hu et al., 2014). In contrast to the observed pattern of soil N degradation gene changes, the abundance of *napB* increased in tandem with irrigation water salinity. This suggests that SWI facilitated the soil DNRA process. Numerous research studies have demonstrated that soil DNRA enhances the conversion of N into NH_4_^+^, serving to safeguard soil N and mitigate the production of N_2_O (Rütting et al., 2011; Bakken et al., 2012). Moreover, the study demonstrated that SWI significantly impacted the abundance of soil nitrification genes (*amoC*), showing a pattern of initially decreasing and then increasing abundance with the increase in irrigation salinity. However, Guo et al. (2023) suggested that soil salinity decreased the abundance of the nitrifying gene *amoC*. The difference may be related to the diverse soil properties. Furthermore, Compared with SWI1, both SWI4 and SWI8 significantly increased denitrifying genes, while SWI4 significantly decreased ANRA genes (*nirA*). This finding aligned with the outcomes of Guo et al. (2023). The surge in denitrification genes could be linked to the adaptation of salt-resistant denitrifying bacteria thriving in high-salinity environments (Magalhães et al., 2005).

### 4.4 Response of microbial taxa in C and N cycling under different levels of irrigation water salinity

Soil contains diverse microbial species with different physiological characteristics (Zhang et al., 2023). Microbial taxa are intricately linked to both the composition and operation of soil ecosystems, playing a vital role in sustaining soil fertility and ecological equilibrium (Jacobsen and Hjelmsø, 2014). In this study, bacteria held a dominant position among soil microorganisms, aligning with the findings of Guo et al. (2023). Additionally, *Actinobacteria* and *Proteobacteria* played a crucial role in both C and N cycling within saltwater-irrigated fields (Supplementary Figures S4, S7). *Actinobacteria* have a significant impact on biogeochemical cycling and soil quality improvement. Wang et al. (2016) showed that *Actinobacteria* was the most abundant halophilic bacteria in saline soils. *Proteobacteria* were recognized as possessing a substantial nutrient utilization capacity and played a pivotal role in steering alterations in soil functionality (van der Bom et al., 2018). Moreover, our results indicated that SWI significantly increased the abundance of *Bacteroidetes* and *Candidatus_Cloacimonetes*. Canfora et al. (2014) also indicated that the abundance of *Bacteroidetes* increased with soil salinity. The changes in soil microbial abundance may be related to soil physical and chemical properties. Guo et al. (2023) suggested that SWI significantly affected soil physical and chemical properties, thus affecting microbial community composition. Moreover, a majority of the microorganisms participating in C and N cycles exhibited similarity at the phylum level (Supplementary Figures S4, S7), suggesting that these predominant microorganisms can be regarded as versatile species with multiple ecological functions (Du et al., 2023). Our results indicated that *Actinobacteria*, *Proteobacteria*, and *Acidobacteria* may be considered general taxa in saltwater-irrigated fields.

### 4.5 C and N cycle processes are intricately interconnected and related to soil properties

Soil salinity, pH, and ammonium N were crucial factors influencing the functional genes and microbial taxa under SWI (Supplementary Figures S9, S10). The effects of soil salinity on functional genes and microbial taxa have been discussed in Sections 4.2, 4.3, and 4.4. It can be seen from Section 3.5 that soil pH significantly affected the process of soil C degradation and fixation in the cotton fields. This could be attributed to the fact that soil pH played the most pivotal role in regulating the potential decomposition rate of stable soil organic C pools (Xiang et al., 2023). Soil pH was also the main factor affecting the microbial function of the soil N cycle. Previous studies have shown that various soil N cycle functional genes have distinct optimal soil pH values (Ouyang et al., 2018). In addition, DNRA process-related genes was significantly positively correlated with soil ammonium N, because it was the product of DNRA (Rütting et al., 2011). The higher the abundance of DNRA-related genes, the more favorable the formation of ammonium N in soil. Moreover, ammonium N was negatively correlated with *amoB*. This may be because the soil nitrification process mainly converts ammonium N into nitrate N (Cui et al., 2016). The rise in nitrification gene abundance could lead to a reduction in soil ammonium content, which serves as the substrate for the soil nitrification process.

In addition to the close correlation between the abundance variation of microbial functional genes and soil properties, functional genes associated with soil C and N cycles tightly interact, either promoting or inhibiting one another, to uphold ecosystem function stability and adapt to shifts in environmental conditions (Luo et al., 2020). Among the top 15 genes in the co-occurrence network, the number in the soil C cycle genes exceeded those in another cycle (N cycle), indicating the importance of C cycle in the saltwater-irrigated cotton fields. The two cycles were mainly connected by DNRA in this study. Pappu et al. (2017) and Zhao et al. (2022) also demonstrated that the process of soil dissimilatory transformation of fixed N (DNRA) tightly combined the N cycle with the C cycle. Heterotrophic prokaryotes involved in the N cycle utilize organic substrates, while autotrophs employ inorganic substrates in their metabolic processes. This interplay establishes a vital connection between the soil N and C cycles (Thamdrup, 2012).

### 4.6 Limitations of this study

In this study, metagenomic methods were used to detect functional genes, which provided information for microbial metabolism mechanisms in the cotton fields under SWI. However, our study should be complemented with transcriptomic information as well as proteomic information of soil microbes to confirm the changes in active microbial metabolism associated with SWI. Future sampling approaches should encompass various growth stages of cotton to evaluate the potential influence of crops on soil function assessment. Experiments should be conducted to analyze the correlation between functional genes and soil microbial metabolites (such as soil CO_2_, N_2_O, and CH_4_), and determine the appropriate salinity of irrigation to ensure the balance of soil C and N cycles.

## 5 Conclusion

This study found that long-term SWI has significantly impacted the microbial functional profile of soil C and N cycles in cotton fields. The data indicates that SWI significantly reduced C degradation gene abundance, which may lead to a decrease in soil C storage. SWI also significantly affects the abundance of functional genes involved in N degradation, DNRA, and nitrification in soil N cycle, which meant that the content of N substrate and microbial metabolites would be changed by SWI. Additionally, similar to previous studies, bacteria such as *Actinobacteria* and *Proteobacteria* occupied the dominant position of microbial taxa in this study. In the cotton fields, changes in the abundance of functional genes and microbial taxa involved in soil C and N cycling were significantly correlated with environmental factors such as soil salinity, pH, and ammonium N content, and there was also evidence of close interconnection between functional gene abundances across soil C and N cycles. This study contributed to exploring the safe utilization of SWI and provided data support for investigating the impact of long-term SWI on the soil C and N cycle microbial functional profile in cotton fields. However, this study only conducted research on soil metagenomics. To comprehensively investigate the microbial impact mechanisms of SWI on soil environment, further integration with soil microbial transcriptomics and proteomics information is necessary.

## Data availability statement

The datasets presented in this study can be found in online repositories. The names of the repository/repositories and accession number(s) can be found at: https://www.ncbi.nlm.nih.gov/, PRJNA1018300.

## Author contributions

SZ: Formal analysis, Investigation, Writing – original draft. GW: Methodology, Writing – review & editing. QH: Methodology, Writing – review & editing. JZ: Supervision, Writing – review & editing. HD: Writing – review & editing. HN: Writing – review & editing. YG: Conceptualization, Methodology, Supervision, Writing – review & editing. JS: Conceptualization, Funding acquisition, Methodology, Supervision, Writing – review & editing.

## Footnotes

^1^ http://www.ncbi.nlm.nih.gov/bioproject/1018300

^2^ https://gephi.org/

## References

[ref1] Bakken L. R. Bergaust L. Liu B. Frostegård Å. (2012). Regulation of denitrification at the cellular level: a clue to the understanding of N2O emissions from soils. Philos. Trans. R. Soc. B Biol. Sci. 367, 1226–1234. doi: 10.1098/rstb.2011.0321, PMID: 22451108 PMC3306626

[ref2] Bernard S. M. Habash D. Z. (2009). The importance of cytosolic glutamine synthetase in nitrogen assimilation and recycling. New Phytol. 182, 608–620. doi: 10.1111/j.1469-8137.2009.02823.x, PMID: 19422547

[ref3] Canfora L. Bacci G. Pinzari F. Lo Papa G. Dazzi C. Benedetti A. (2014). Salinity and bacterial diversity: to what extent does the concentration of salt affect the bacterial community in a saline soil? PLoS One 9:e106662. doi: 10.1371/journal.pone.0106662, PMID: 25188357 PMC4154724

[ref4] Cao S. K. Chen K. L. Cao G. C. Zhang L. Ma J. Yang L.. (2011). The analysis of characteristic and spatial variability or soil organic matter and organic carbon around Qinghai Lake. Procedia Environ. Sci. 10, 678–684. doi: 10.1016/j.proenv.2011.09.109

[ref5] Cui Y.-W. Zhang H.-Y. Ding J.-R. Peng Y.-Z. (2016). The effects of salinity on nitrification using halophilic nitrifiers in a sequencing batch reactor treating hypersaline wastewater. Sci. Rep. 6:24825. doi: 10.1038/srep24825, PMID: 27109617 PMC4843016

[ref6] Dixon N. E. Gazzola C. Blakeley R. L. Zerner B. (1976). Metal ions in enzymes using ammonia or amides. Science 191, 1144–1150. doi: 10.1126/science.769157 769157

[ref7] Du T. Hu Q. Mao W. Yang Z. Chen H. Sun L.. (2023). Metagenomics insights into the functional profiles of soil carbon, nitrogen, and phosphorus cycles in a walnut orchard under various regimes of long-term fertilisation. Eur. J. Agron. 148:126887. doi: 10.1016/j.eja.2023.126887

[ref8] Gao W. Cheng S. Fang H. Chen Y. Yu G. Zhou M.. (2013). Effects of simulated atmospheric nitrogen deposition on inorganic nitrogen content and acidification in a cold-temperate coniferous forest soil. Acta Ecol. Sin. 33, 114–121. doi: 10.1016/j.chnaes.2013.01.008

[ref9] Geisseler D. Horwath W. R. Joergensen R. G. Ludwig B. (2010). Pathways of nitrogen utilization by soil microorganisms - a review. Soil Biol. Biochem. 42, 2058–2067. doi: 10.1016/j.soilbio.2010.08.021

[ref10] Guo X. Du S. Guo H. Min W. (2023). Long-term saline water drip irrigation alters soil physicochemical properties, bacterial community structure, and nitrogen transformations in cotton. Appl. Soil Ecol. 182:104719. doi: 10.1016/j.apsoil.2022.104719

[ref11] Haj-Amor Z. Araya T. Kim D.-G. Bouri S. Lee J. Ghiloufi W.. (2022). Soil salinity and its associated effects on soil microorganisms, greenhouse gas emissions, crop yield, biodiversity and desertification: a review. Sci. Total Environ. 843:156946. doi: 10.1016/j.scitotenv.2022.156946, PMID: 35768029

[ref12] Han X. Li D. Kang Y. Wan S. (2023). Effect of saline water drip irrigation on tomato yield and quality in semi-humid area and arid area of China. Irrig. Sci. 42, 387–400. doi: 10.1007/s00271-023-00870-x

[ref13] Hu X. Gu H. Liu J. Wei D. Zhu P. Cui X.. (2022). Metagenomics reveals divergent functional profiles of soil carbon and nitrogen cycling under long-term addition of chemical and organic fertilizers in the black soil region. Geoderma 418:115846. doi: 10.1016/j.geoderma.2022.115846

[ref14] Hu Y. Wang L. Tang Y. Li Y. Chen J. Xi X.. (2014). Variability in soil microbial community and activity between coastal and riparian wetlands in the Yangtze River estuary – potential impacts on carbon sequestration. Soil Biol. Biochem. 70, 221–228. doi: 10.1016/j.soilbio.2013.12.025

[ref15] Jacobsen C. S. Hjelmsø M. H. (2014). Agricultural soils, pesticides and microbial diversity. Curr. Opin. Biotechnol. 27, 15–20. doi: 10.1016/j.copbio.2013.09.003 24863892

[ref16] Jing H. Li J. Yan B. Wei F. Wang G. Liu G. (2021). The effects of nitrogen addition on soil organic carbon decomposition and microbial C-degradation functional genes abundance in a Pinus tabulaeformis forest. For. Ecol. Manag. 489:119098. doi: 10.1016/j.foreco.2021.119098

[ref17] Lawson C. E. Wu S. Bhattacharjee A. S. Hamilton J. J. McMahon K. D. Goel R.. (2017). Metabolic network analysis reveals microbial community interactions in anammox granules. Nat. Commun. 8:15416. doi: 10.1038/ncomms15416, PMID: 28561030 PMC5460018

[ref18] Lew B. Tarnapolski O. Afgin Y. Portal Y. Ignat T. Yudachev V.. (2020). Exploratory ranking analysis of brackish groundwater desalination for sustainable agricultural production: a case study of the Arava Valley in Israel. J. Arid Environ. 174:104078. doi: 10.1016/j.jaridenv.2019.104078

[ref19] Li X.-R. Xiao Y.-P. Ren W.-W. Liu Z.-F. Shi J.-H. Quan Z. X. (2012). Abundance and composition of ammonia-oxidizing bacteria and archaea in different types of soil in the Yangtze River estuary. J Zhejiang Univ Sci B 13, 769–782. doi: 10.1631/jzus.B1200013, PMID: 23024044 PMC3468820

[ref20] Loganathachetti D. Alhashmi F. Chandran S. Mundra S. (2022). Irrigation water salinity structures the bacterial communities of date palm (*Phoenix dactylifera*)-associated bulk soil. Front. Plant Sci. 13:944637. doi: 10.3389/fpls.2022.944637, PMID: 35991423 PMC9388049

[ref21] Luo R. Fan J. Wang W. Luo J. Kuzyakov Y. He J.-S.. (2019). Nitrogen and phosphorus enrichment accelerates soil organic carbon loss in alpine grassland on the Qinghai-Tibetan plateau. Sci. Total Environ. 650, 303–312. doi: 10.1016/j.scitotenv.2018.09.038, PMID: 30199676

[ref22] Luo G. Xue C. Jiang Q. Xiao Y. Zhang F. Guo S.. (2020). Soil carbon, nitrogen, and phosphorus cycling microbial populations and their resistance to global change depend on soil C:N:P stoichiometry. mSystems 5:e00162-20. doi: 10.1128/mSystems.00162-20, PMID: 32606023 PMC7329320

[ref23] Magalhães C. M. Joye S. B. Moreira R. M. Wiebe W. J. Bordalo A. A. (2005). Effect of salinity and inorganic nitrogen concentrations on nitrification and denitrification rates in intertidal sediments and rocky biofilms of the Douro River estuary, Portugal. Water Res. 39, 1783–1794. doi: 10.1016/j.watres.2005.03.008, PMID: 15899276

[ref24] Mamilov A. Dilly O. M. Mamilov S. Inubushi K. (2004). Microbial eco-physiology of degrading Aral Sea wetlands: consequences for C-cycling. Soil Sci. Plant Nutr. 50, 839–842. doi: 10.1080/00380768.2004.10408544

[ref25] Morrissey E. M. Gillespie J. L. Morina J. C. Franklin R. B. (2014). Salinity affects microbial activity and soil organic matter content in tidal wetlands. Glob. Chang. Biol. 20, 1351–1362. doi: 10.1111/gcb.12431, PMID: 24307658

[ref26] Ouyang Y. Evans S. E. Friesen M. L. Tiemann L. K. (2018). Effect of nitrogen fertilization on the abundance of nitrogen cycling genes in agricultural soils: a meta-analysis of field studies. Soil Biol. Biochem. 127, 71–78. doi: 10.1016/j.soilbio.2018.08.024

[ref27] Pappu A. R. Bhattacharjee A. S. Dasgupta S. Goel R. (2017). Nitrogen cycle in engineered and natural ecosystems—past and current. Curr. Pollut. Rep. 3, 120–140. doi: 10.1007/s40726-017-0051-y

[ref28] Poffenbarger H. J. Needelman B. A. Megonigal J. P. (2011). Salinity influence on methane emissions from tidal marshes. Wetlands 31, 831–842. doi: 10.1007/s13157-011-0197-0

[ref29] Ren C. Wang J. Bastida F. Delgado-Baquerizo M. Yang Y. Wang J.. (2022). Microbial traits determine soil C emission in response to fresh carbon inputs in forests across biomes. Glob. Chang. Biol. 28, 1516–1528. doi: 10.1111/gcb.16004, PMID: 34807491

[ref30] Rütting T. Boeckx P. Müller C. Klemedtsson L. (2011). Assessment of the importance of dissimilatory nitrate reduction to ammonium for the terrestrial nitrogen cycle. Biogeosciences 8, 1779–1791. doi: 10.5194/bg-8-1779-2011

[ref31] Saviozzi A. Cardelli R. Di Puccio R. (2011). Impact of salinity on soil biological activities: a laboratory experiment. Commun. Soil Sci. Plant Anal. 42, 358–367. doi: 10.1080/00103624.2011.542226

[ref32] Shah T. Lateef S. Noor M. A. (2020). “Carbon and nitrogen cycling in agroecosystems: an overview” in Carbon and Nitrogen Cycling in Soil, 1–15.

[ref33] Shahariar S. Farrell R. Soolanayakanahally R. Bedard-Haughn A. (2021). Elevated salinity and water table drawdown significantly affect greenhouse gas emissions in soils from contrasting land-use practices in the prairie pothole region. Biogeochemistry 155, 127–146. doi: 10.1007/s10533-021-00818-3

[ref34] Shen C. Shi Y. Ni Y. Deng Y. Van Nostrand J. He Z.. (2016). Dramatic increases of soil microbial functional gene diversity at the Treeline ecotone of Changbai Mountain. Front. Microbiol. 7:1184. doi: 10.3389/fmicb.2016.01184, PMID: 27524983 PMC4965465

[ref35] Shi X. Zhou Y. Zhao X. Guo P. Ren J. Zhang H.. (2023). Soil metagenome and metabolome of peanut intercropped with sorghum reveal a prominent role of carbohydrate metabolism in salt-stress response. Environ. Exp. Bot. 209:105274. doi: 10.1016/j.envexpbot.2023.105274

[ref36] Sun R. Wang F. Hu C. Liu B. (2021). Metagenomics reveals taxon-specific responses of the nitrogen-cycling microbial community to long-term nitrogen fertilization. Soil Biol. Biochem. 156:108214. doi: 10.1016/j.soilbio.2021.108214

[ref37] Suter E. A. Pachiadaki M. G. Montes E. Edgcomb V. P. Scranton M. I. Taylor C. D.. (2021). Diverse nitrogen cycling pathways across a marine oxygen gradient indicate nitrogen loss coupled to chemoautotrophic activity. Environ. Microbiol. 23, 2747–2764. doi: 10.1111/1462-2920.15187 32761757

[ref38] Tang X. Xie G. Shao K. Bayartu S. Chen Y. Gao G. (2012). Influence of salinity on the bacterial community composition in Lake Bosten, a large oligosaline lake in arid northwestern China. Appl. Environ. Microbiol. 78, 4748–4751. doi: 10.1128/AEM.07806-11, PMID: 22522679 PMC3370503

[ref39] Thamdrup B. (2012). New pathways and processes in the global nitrogen cycle. Annu. Rev. Ecol. Evol. Syst. 43, 407–428. doi: 10.1146/annurev-ecolsys-102710-145048

[ref40] van der Bom F. Nunes I. Raymond N. S. Hansen V. Bonnichsen L. Magid J.. (2018). Long-term fertilisation form, level and duration affect the diversity, structure and functioning of soil microbial communities in the field. Soil Biol. Biochem. 122, 91–103. doi: 10.1016/j.soilbio.2018.04.003

[ref41] Wang H. Feng D. Zhang A. Zheng C. Li K. Ning S.. (2022). Effects of saline water mulched drip irrigation on cotton yield and soil quality in the North China plain. Agric. Water Manag. 262:107405. doi: 10.1016/j.agwat.2021.107405

[ref42] Wang H. Gilbert J. A. Zhu Y. Yang X. (2018). Salinity is a key factor driving the nitrogen cycling in the mangrove sediment. Sci. Total Environ. 631-632, 1342–1349. doi: 10.1016/j.scitotenv.2018.03.102, PMID: 29727958

[ref43] Wang Z. Luo G. Li J. Chen S.-Y. Li Y. Li W.-T.. (2016). Response of performance and ammonia oxidizing bacteria community to high salinity stress in membrane bioreactor with elevated ammonia loading. Bioresour. Technol. 216, 714–721. doi: 10.1016/j.biortech.2016.05.123, PMID: 27290667

[ref44] Xiang D. Wang G. Tian J. Li W. (2023). Global patterns and edaphic-climatic controls of soil carbon decomposition kinetics predicted from incubation experiments. Nat. Commun. 14:2171. doi: 10.1038/s41467-023-37900-3, PMID: 37061518 PMC10105724

[ref45] Yan N. Marschner P. Cao W. Zuo C. Qin W. (2015). Influence of salinity and water content on soil microorganisms. Int. Soil Water Conserv. Res. 3, 316–323. doi: 10.1016/j.iswcr.2015.11.003

[ref46] Yang C. Chen Y. Zhang Q. Qie X. Chen J. Che Y.. (2023). Mechanism of microbial regulation on methane metabolism in saline–alkali soils based on metagenomics analysis. J. Environ. Manag. 345:118771. doi: 10.1016/j.jenvman.2023.118771, PMID: 37591100

[ref47] Yu Y. Zhao C. Zheng N. Jia H. Yao H. (2019). Interactive effects of soil texture and salinity on nitrous oxide emissions following crop residue amendment. Geoderma 337, 1146–1154. doi: 10.1016/j.geoderma.2018.11.012

[ref48] ZhangG.BaiJ.ZhaiY.JiaJ.ZhaoQ.WangW.. (2023). Microbial diversity and functions in saline soils: a review from a biogeochemical perspective. J. Adv. Res. doi: 10.1016/j.jare.2023.06.015. Epub ahead of print. PMID: 37392974

[ref49] Zhang L. Jing Y. Chen C. Xiang Y. Rezaei Rashti M. Li Y.. (2021). Effects of biochar application on soil nitrogen transformation, microbial functional genes, enzyme activity, and plant nitrogen uptake: a meta-analysis of field studies. GCB Bioenergy 13, 1859–1873. doi: 10.1111/gcbb.12898

[ref50] Zhang A. Zheng C. Li K. Dang H. Cao C. Rahma A. E.. (2020). Responses of soil water-salt variation and cotton growth to drip irrigation with saline water in the low plain near the BOHAI Sea. Irrig. Drain. 69, 448–459. doi: 10.1002/ird.2428

[ref51] Zhao Y. Li Q. Cui Q. Ni S.-Q. (2022). Nitrogen recovery through fermentative dissimilatory nitrate reduction to ammonium (DNRA): carbon source comparison and metabolic pathway. Chem. Eng. J. 441:135938. doi: 10.1016/j.cej.2022.135938

[ref52] Zhou S. Gao Y. Zhang J. Pang J. Hamani A. K. Xu C.. (2023). Impacts of saline water irrigation on soil respiration from cotton fields in the North China plain. Agronomy 13:1197. doi: 10.3390/agronomy13051197

[ref53] Zhou S. Wang G. Zhang J. Dang H. Gao Y. Sun J. (2024). Long-term saline water irrigation has the potential to balance greenhouse gas emissions and cotton yield in North China plain. J. Environ. Manag. 352:120087. doi: 10.1016/j.jenvman.2024.120087, PMID: 38215592

