# Supplementary material for: Long-term saline water irrigation affected soil carbon and nitrogen cycling functional profiles in the cotton field
Source: Front Microbiol. 2024 Mar 14;15:1310387. doi: 10.3389/fmicb.2024.1310387 (PMC10972904; doi:10.3389/fmicb.2024.1310387)
Supplement: Supplementary file 1 [file Data_Sheet_1.docx]

Supplementary Material

# Supplementary text 1

Analysis of soil properties:

(1) Soil Salinity (EC*_e_*)：

After air-drying, the soil samples were ground and then sieved using a 1 mm sieve. The electrical conductivity (EC_1:5_) of the mixed solution, prepared using soil and water in a 1:5 ratio by weight, was measured using a conductivity meter (Orion Star A322, Thermo Scientific, USA). To facilitate comparisons with other international research, the EC of the saturated soil extract (EC*_e_*) was also measured. The saturated paste was prepared according to the US Salinity Laboratory (1954) method, which was employed to obtain a saturated extract. EC*_e_* was measured using the conductivity meter, and the relationship between the EC_1:5_ and EC*_e_* is expressed by the following formula:

| $\text{EC}_{\text{e}}\text{ = 9.367}\text{EC}_{\text{1:5}}\text{ - 0.001}$,  $\text{R}^{\text{2}}\text{ = 0.990}$ |  |
| --- | --- |

(2) pH：

The pH probe (Orion Star A321, Thermo Scientific, USA) was used for measurement.

(3) Soil nitrate nitrogen and ammonium nitrogen：

The fresh soil was weighed to 10 g and extracted with KCL solution. The contents of soil nitrate nitrogen and ammonium nitrogen were measured by a flow analyzer (Thermo, VarioskanFlash, USA).

(4) Soil organic matter：

Soil organic matter was determined by the low-temperature external thermal potassium dichromate oxidation-colorimetric method. The soil samples were air-dried through a 0.15mm sieve, and then heated in a water bath with potassium dichromate solution and concentrated sulfuric acid. The absorbance was measured by an ultraviolet-visible spectrophotometer (UV-1200, Mepda, Shanghai). Calculate the content of soil organic matter according to the formula below.

$$\text{OM=}\frac{\text{m}_{\text{1}}\text{×1.724×1.08}}{\text{m×100}}\times\text{1000}$$

where OM (g·kg^-1^) is soil organic matter content. m_1_ (mg) is soil carbon content obtained from the absorption value against the standard curve. m (g) is the weight of the soil. 1.724 is the conversion coefficient of soil organic carbon to organic matter (calculated as the average carbon content of soil organic matter is 58%). 1.08 is an oxidation correction factor.

# Supplementary Figures and Tables

## Supplementary Figures

**Figure S1.** Relative abundance ratio of functional genes involved in soil carbon cycling in saline irrigated cotton fields. Functional genes marked with asterisks differ significantly in the proportion of gene abundance in multiple group comparisons.

Note: C is carbon. SWI1: irrigation water salinity was 1 g L^-1^. SWI4: irrigation water salinity was 4 g L^-1^. SWI8: irrigation water salinity was 8 g L^-1^.

**Figure S2.** Relative abundance ratio of functional genes involved in soil nitrogen cycling in saline irrigated cotton fields. Functional genes marked with asterisks that differ significantly in the proportion of gene abundance in multiple group comparison

Note: N is nitrogen. SWI1: irrigation water salinity was 1 g L^-1^. SWI4: irrigation water salinity was 4 g L^-1^. SWI8: irrigation water salinity was 8 g L^-1^.

**Figure S3.** The relative abundance of microbial taxa (> 0.1%) by carbon cycling genes at the phylum.

Note: SWI1: irrigation water salinity was 1 g L^-1^. SWI4: irrigation water salinity was 4 g L^-1^. SWI8: irrigation water salinity was 8 g L^-1^.

**Figure4.** The comparison of microbial taxa (> 0.1%) by carbon cycling between different saline water irrigation treatments. Significance levels of each predictor are “*” p<0.05, “**” p<0.01, “***” p<0.001.

Note: SWI1: irrigation water salinity was 1 g L^-1^. SWI4: irrigation water salinity was 4 g L^-1^. SWI8: irrigation water salinity was 8 g L^-1^.

**Figure S5.** Carbon cycling microbial taxa (> 0.1%) were significantly affected by saline water irrigation

Note: SWI1: irrigation water salinity was 1 g L^-1^. SWI4: irrigation water salinity was 4 g L^-1^. SWI8: irrigation water salinity was 8 g L^-1^.

**Figure S6.** The relative abundance of microbial taxa (> 0.1%) by nitrogen cycling genes at the phylum.

**Figure S7.** The comparison of microbial taxa (> 0.1%) by nitrogen cycling between different saline water irrigation treatments. Significance levels of each predictor are “*” p<0.05, “**” p<0.01, “***” p<0.001.

Note: N is nitrogen. SWI1: irrigation water salinity was 1 g L^-1^. SWI4: irrigation water salinity was 4 g L^-1^. SWI8: irrigation water salinity was 8 g L^-1^.

**Figure S8.** Nitrogen cycling microbial taxa (> 0.1%) were significantly affected by saline water irrigation

Note: SWI1: irrigation water salinity was 1 g L^-1^. SWI4: irrigation water salinity was 4 g L^-1^. SWI8: irrigation water salinity was 8 g L^-1^.

**Figure S9.** Correlation heat maps of soil properties and abundance of C and N cycle function genes in saline irrigated cotton fields. “*” p<0.05, “**” p<0.01, “***” p<0.001.


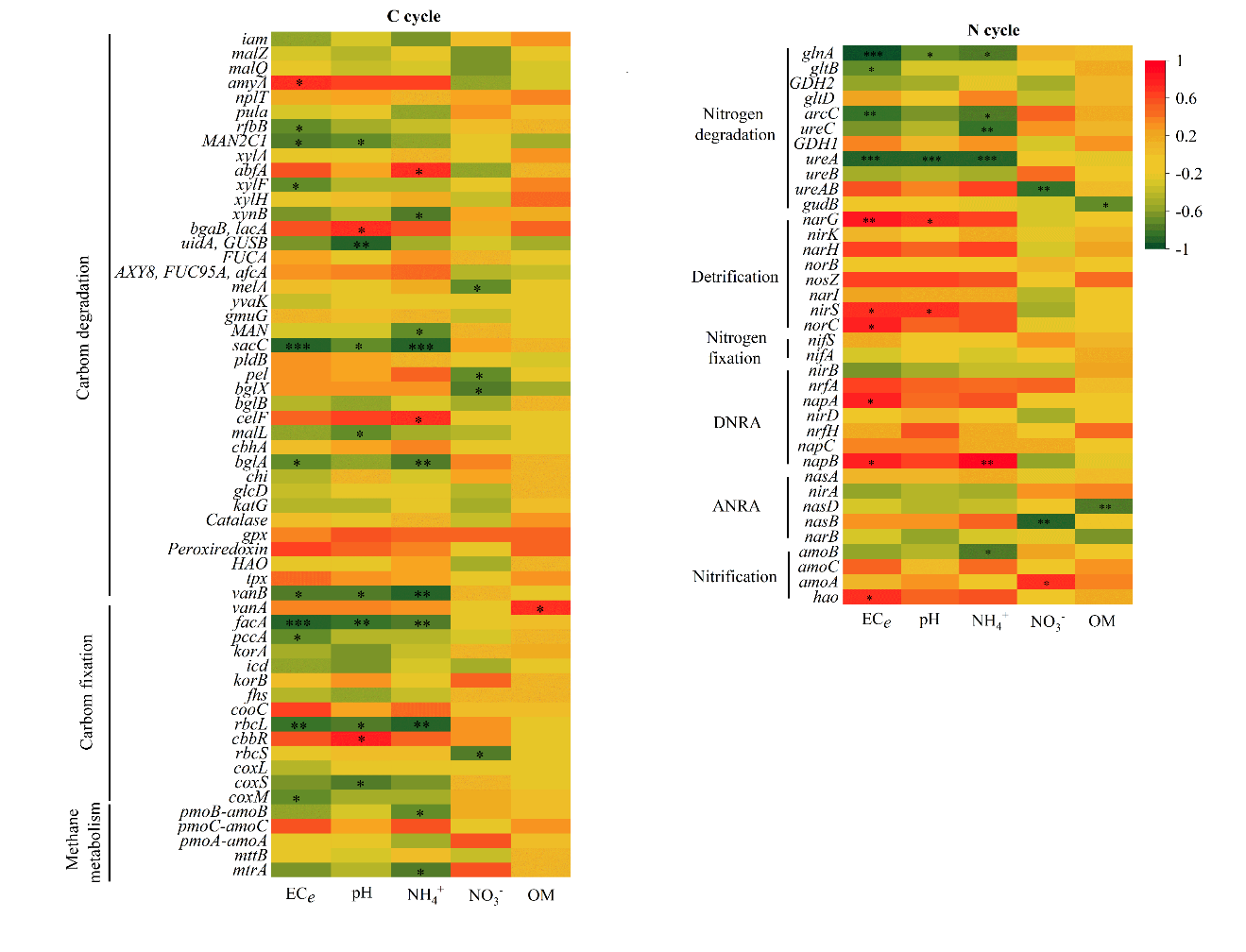


Note: C is carbon. N is nitrogen. EC*_e_* = electrical conductivity of a saturated soil extract. NH_4_^+^ = nitrate nitrogen. NO_3_^-^ = ammonium nitrogen. OM = organic matter.

**Figure S10.** Correlation heat maps of soil properties and microbial taxa of C and N cycle in saline irrigated cotton fields. “*” p<0.05, “**” p<0.01, “***” p<0.001.

Note: C is carbon. N is nitrogen. EC*_e_* = electrical conductivity of a saturated soil extract. NH_4_^+^ = nitrate nitrogen. NO_3_^-^ = ammonium nitrogen. OM = organic matter.

**Table S1** Ion composition of the irrigation water.

| Treat-  ments | Salinity of irrigation water  (g L^-1^) | Ion concentration (mEq L^-1^) | | | | | | |
| --- | --- | --- | --- | --- | --- | --- | --- | --- |
|  |  | Ca^2+^ | Mg^2+^ | K^+^ | Na^+^ | SO_4_^2-^ | HCO_3_^-^ | CL^-^ |
| SWI1 | 1 | 1.43 | 1.61 | 0.15 | 10.73 | 5.88 | 1.04 | 7.36 |
| SWI4 | 4 | 2.00 | 4.60 | 0.20 | 56.44 | 16.91 | 1.21 | 47.27 |
| SWI8 | 8 | 2.79 | 6.26 | 0.25 | 119.97 | 30.14 | 1.44 | 100.04 |

Note: SWI1: irrigation water salinity was 1 g L^-1^. SWI4: irrigation water salinity was 4 g L^-1^. SWI8: irrigation water salinity was 8 g L^-1^.

**Table S2** Basic information on metagenomic sequencing

| Treatments | Samples | Clean reads | Percent in raw reads (%) | Contigs | ORFs | N50(bp) | N90(bp) |
| --- | --- | --- | --- | --- | --- | --- | --- |
| SWI1 | Z1 | 50184796 | 97.75737 | 448996 | 522099 | 490 | 330 |
| SWI1 | Z4 | 47090048 | 97.68872 | 421159 | 487876 | 490 | 330 |
| SWI1 | Z9 | 53174852 | 97.36288 | 505128 | 594467 | 513 | 334 |
| SWI4 | Z2 | 49463838 | 97.53823 | 480483 | 559796 | 495 | 331 |
| SWI4 | Z5 | 44084282 | 96.80806 | 414591 | 483523 | 502 | 332 |
| SWI4 | Z7 | 56108858 | 97.35932 | 628053 | 746619 | 522 | 335 |
| SWI8 | Z3 | 45888936 | 96.76095 | 527481 | 620508 | 506 | 333 |
| SWI8 | Z6 | 46989420 | 97.47074 | 577413 | 685123 | 519 | 335 |
| SWI8 | Z8 | 43901592 | 97.52717 | 426249 | 515127 | 563 | 337 |

Note: Percent in raw reads is the percentage of clean read in its corresponding raw read. ORFs, Open reading frames. N50 represents the length of the contig overlapping the midpoint of the length-order concatenation of contigs. N90 represents the length of the contig overlapping the ninety percent of the length-order concatenation of contigs.

**Table S3** Information of microbial functional genes involved in the C and N cycling processes identified in this study.

| KEGG orthologty number | Gene name | Encoded prottein (EC)/KO Description |
| --- | --- | --- |
| **C cycle** |  |  |
| **C degradation** |  |  |
| **Starch** |  |  |
| K00705 | *malQ* | 4-alpha-glucanotransferase [EC:2.4.1.25] |
| K01176 | *amyA* | alpha-amylase [EC:3.2.1.1] |
| K01187 | *malZ* | alpha-glucosidase [EC:3.2.1.20] |
| K01200 | *pula* | pullulanase [EC:3.2.1.41] |
| K01208 | *nplT* | neopullulanase [EC:3.2.1.135] |
| K01214 | *iam* | isoamylase [EC:3.2.1.68] |
| **Hemicellulose** |  |  |
| K01048 | *pldB* | lysophospholipase [EC:3.1.1.5] |
| K01191 | *MAN2C1* | alpha-mannosidase [EC:3.2.1.24] |
| K01195 | *uidA, GUSB* | beta-glucuronidase [EC:3.2.1.31] |
| K01198 | *xynB* | xylan 1,4-beta-xylosidase [EC:3.2.1.37] |
| K01206 | *FUCA* | alpha-L-fucosidase [EC:3.2.1.51] |
| K01209 | *abfA* | alpha-L-arabinofuranosidase [EC:3.2.1.55] |
| K01212 | *sacC* | levanase [EC:3.2.1.65] |
| K01218 | *gmuG* | mannan endo-1,4-beta-mannosidase [EC:3.2.1.78] |
| K01710 | *rfbB* | phosphomannomutase [EC:4.2.1.46] |
| K01805 | *xylA* | xylose isomerase [EC:5.3.1.5] |
| K03928 | *yvaK* | carboxylesterase [EC:3.1.1.1] |
| K07406 | *melA* | alpha-galactosidase [EC:3.2.1.22] |
| K10543 | *xylF* | D-xylose transporter |
| K10544 | *xylH* | D-xylose transport system permease protein |
| K12308 | *bgaB, lacA* | beta-galactosidase [EC:3.2.1.23] |
| K15923 | *AXY8, FUC95A, afcA* | alpha-L-fucosidase 2 [EC:3.2.1.51] |
| K19355 | *MAN* | mannan endo-1,4-beta-mannosidase [EC:3.2.1.78] |
| **Pectin** |  |  |
| K01728 | *pel* | pectate lyase [EC:4.2.2.2] |
| **Cellulose** |  |  |
| K01179 | *celF* | endoglucanase F [EC:3.2.1.4] |
| K01223 | *bglA* | 6-phospho-beta-glucosidase [EC:3.2.1.86] |
| K05349 | *bglX* | beta-glucosidase [EC:3.2.1.21] |
| K05350 | *bglB* | beta-glucosidase [EC:3.2.1.21] |
| K19668 | *cbhA* | 1,4-beta-cellobiohydrolase A [EC:3.2.1.91] |
| K01182 | *malL* | oligo-1,6-glucosidase [EC:3.2.1.10] |
| **Chitin** |  |  |
| K01183 | *chi* | chitinase [EC:3.2.1.14] |
| **Lignin** |  |  |
| K00104 | *glcD* | glycolate dehydrogenase FAD-linked subunit [EC:1.1.99.14] |
| K00432 | *gpx* | glutathione peroxidase [EC:1.11.1.9] |
| K03386 | *Peroxiredoxin* | peroxiredoxin 2/4 [EC:1.11.1.24] |
| K03781 | *Catalase* | catalase [EC:1.11.1.6] |
| K03782 | *katG* | catalase-peroxidase [EC:1.11.1.21] |
| K03862 | *vanA* | vanillate monooxygenase [EC:1.14.13.82] |
| K03863 | *vanB* | vanillate monooxygenase ferredoxin subunit |
| K11065 | *tpx* | thioredoxin-dependent peroxiredoxin [EC:1.11.1.24] |
| K11517 | *HAO* | (S)-2-hydroxy-acid oxidase [EC:1.1.3.15] |
| **C fixation** |  |  |
| **Multiple systems** |  |  |
| K11263 | *pccA* | propionyl-CoA carboxylase [EC:6.4.1.3] |
| K01895 | *facA* | acetyl-CoA synthetase [EC:6.2.1.1] |
| **Reductive tricarboxylic acid cycle (rTCA cycle)** | | |
| K00031 | *icd* | isocitrate dehydrogenase [EC:1.1.1.42] |
| K00174 | *korA* | 2-oxoglutarate ferredoxin oxidoreductase subunit alpha [EC:1.2.7.11] |
| K00175 | *korB* | 2-oxoglutarate ferredoxin oxidoreductase subunit beta [EC:1.2.7.11] |
| **Reductive acetyl-CoA pathway** | | |
| K01938 | *fhs* | formyltetrahydrofolate synthetase [EC:6.3.4.3] |
| K07321 | *cooC* | carbon monoxide dehydrogenase accessory protein |
| **Calvin cycle** |  |  |
| K01601 | *rbcL* | ribulose bisphosphate carboxylase large chain [EC:4.1.1.39] |
| K01602 | *rbcS* | ruBisCO small subunit [EC:4.1.1.39] |
| K21703 | *cbbR* | ruBisCO operon transcriptional regulator |
| **CO oxidation** |  |  |
| K03518 | *coxS* | carbon monoxide dehydrogenase small subunit [EC:1.2.5.3] |
| K03519 | *coxM* | carbon monoxide dehydrogenase medium subunit [EC:1.2.5.3] |
| K03520 | *coxL* | carbon monoxide dehydrogenase large subunit [EC:1.2.5.3] |
| **Methane metabolism** | |  |
| **Methane oxidation** | |  |
| K10944 | *pmoA-amoA* | methane/ammonia monooxygenase subunit A [EC:1.14.18.3, 1.14.99.39] |
| K10945 | *pmoB-amoB* | methane/ammonia monooxygenase subunit B |
| K10946 | *pmoC-amoC* | methane/ammonia monooxygenase subunit C |
| **Methanogen** |  |  |
| K00577 | *mtrA* | N5-methyltetrahydromethanopterin-coenzyme M methyltransferase subunit A [EC:2.1.1.86] |
| K14083 | *mttB* | trimethylamine methyltransferase [EC:2.1.1.250] |
|  |  |  |
| **N cycle** |  |  |
| **Nitrogen fixation** |  |  |
| K02584 | *nifA* | nif-specific regulatory protein |
| K04487 | *nifS* | Nitrogenase metalloclusters biosynthesis protein [EC:2.8.1.7] |
| **Nitrification** |  |  |
| K10944 | *amoA-pmoA* | ammonia monooxygenase subunit A/methane [EC:1.14.18.3 1.14.99.39] |
| K10945 | *amoB-pmoB* | ammonia monooxygenase subunit B/methane |
| K10946 | *amoC-pmoC* | ammonia monooxygenase subunit C/methane |
| K10535 | *hao* | hydroxylamine dehydrogenase [EC:1.7.2.6] |
| **Detrification** |  |  |
| K00368 | *nirK* | nitrite reductase (NO-forming) [EC:1.7.2.1] |
| K00370 | *narG* | nitrate reductase / nitrite oxidoreductase, alpha subunit [EC:1.7.5.1 1.7.99.-] |
| K00371 | *narH* | nitrate reductase / nitrite oxidoreductase, beta subunit [EC:1.7.5.1 1.7.99.-] |
| K00374 | *narI* | nitrate reductase gamma subunit [EC:1.7.5.1 1.7.99.-] |
| K00376 | *nosZ* | nitrous-oxide reductase [EC:1.7.2.4] |
| K02305 | *norC* | nitric oxide reductase subunit C |
| K04561 | *norB* | nitric oxide reductase subunit B [EC:1.7.2.5] |
| K15864 | *nirS* | nitrite reductase (NO-forming) / hydroxylamine reductase [EC:1.7.2.1 1.7.99.1] |
| **Assimilatory nitrate reduction (ANRA)** | | |
| K00360 | *nasB* | assimilatory nitrate reductase electron transfer subunit [EC:1.7.99.-] |
| K00366 | *nirA* | ferredoxin-nitrite reductase [EC:1.7.7.1] |
| K00367 | *narB* | ferredoxin-nitrate reductase [EC:1.7.7.2] |
| K00372 | *nasA* | assimilatory nitrate reductase catalytic subunit [EC:1.7.99.-] |
| K15578 | *nasD* | nitrate/nitrite transport system ATP-binding protein [EC:7.3.2.4] |
| **Dissimilatory nitrate reduction (DNRA)** | | |
| K00362 | *nirB* | nitrite reductase (NADH) large subunit [EC:1.7.1.15] |
| K00363 | *nirD* | nitrite reductase (NADH) small subunit [EC:1.7.1.15] |
| K02567 | *napA* | nitrate reductase (cytochrome) [EC:1.9.6.1] |
| K02568 | *napB* | nitrate reductase (cytochrome), electron transfer subunit |
| K02569 | *napC* | periplasmic nitrate reductase, electron transfer subunit |
| K03385 | *nrfA* | nitrite reductase (cytochrome c-552) [EC:1.7.2.2] |
| K15876 | *nrfH* | cytochrome c nitrite reductase small subunit |
| **Nitrogen degradation** |  |  |
| K00260 | *gudB* | glutamate dehydrogenase [EC:1.4.1.2] |
| K00262 | *GDH1* | NADP-specific glutamate dehydrogenase [EC:1.4.1.4] |
| K01428 | *ureC* | urease subunit beta [EC:3.5.1.5] |
| K01429 | *ureB* | Urease subunit alpha [EC:3.5.1.5] |
| K01430 | *ureA* | urease subunit gamma |
| K14048 | *ureAB* | urease subunit gamma/beta [EC:3.5.1.5] |
| K01915 | *glnA* | glutamine synthetase [EC:6.3.1.2] |
| K00265 | *gltB* | glutamate synthase (NADPH) large chain [EC:1.4.1.13] |
| K00266 | *gltD* | glutamate synthase (NADPH) small chain [EC:1.4.1.13] |
| K00926 | *arcC* | carbamate kinase [EC:2.7.2.2] |
| K15371 | *GDH2* | NAD-specific glutamate dehydrogenase [EC:1.4.1.2] |

**Table S4** Soil properties of the cotton field under long-term saline irrigation

| Treatments | EC*_e_* | pH | NH_4_^+^ | NO_3_^-^ | OM |
| --- | --- | --- | --- | --- | --- |
| SWI1 | 2.06 | 7.90 | 0.82 | 86.66 | 14.59 |
| SWI4 | 3.75 | 8.11 | 0.86 | 83.89 | 13.91 |
| SWI8 | 7.65 | 8.23 | 2.04 | 78.41 | 13.97 |

Note: EC*_e_* = electrical conductivity of a saturated soil extract. NH_4_^+^ = nitrate nitrogen. NO_3_^-^ = ammonium nitrogen. OM = organic matter.
